# Lactose: Characteristics, Food and Drug-Related Applications, and Its Possible Substitutions in Meeting the Needs of People with Lactose Intolerance

**DOI:** 10.3390/foods11101486

**Published:** 2022-05-19

**Authors:** Simona Dominici, Francesca Marescotti, Chiara Sanmartin, Monica Macaluso, Isabella Taglieri, Francesca Venturi, Angela Zinnai, Maria Sole Facioni

**Affiliations:** 1 ELLEFREE S.r.l., Polo Tecnologico Lucchese, 55100 Lucca, Italy; sdominici@ellefree.com (S.D.); qualita@ellefree.com (F.M.); 2 Department of Agriculture, Food and Environment, University of Pisa, Via del Borghetto 80, 56124 Pisa, Italy; chiara.sanmartin@unipi.it (C.S.); monica.macaluso@phd.unipi.it (M.M.); francesca.venturi@unipi.it (F.V.); angela.zinnai@unipi.it (A.Z.); 3 Interdepartmental Research Center “Nutraceuticals and Food for Health”, University of Pisa, Via del Borghetto 80, 56124 Pisa, Italy; 4 AILI—Associazione Italiana Latto-Intolleranti Aps, 55100 Lucca, Italy; presidente@associazioneaili.it

**Keywords:** lactose, chemical properties, food application, excipient, legislation, lactose intolerance, lactose-free

## Abstract

The recent growing interest in lactose intolerance has resulted in the proliferation of lactose-free products by food manufacturing companies. Since updated papers about lactose and its uses are missing, the main purpose of this review is to investigate this sugar comprehensively. Firstly, its chemical and physical characteristics were studied, following its employment in the food and drug industries. The positive and negative health-related effects of lactose are reported, focusing on the condition of lactose intolerance, for which an adequate lactose-free diet has to be followed to avoid symptoms that impairs quality of life. Considering that EU legislation on lactose-free product labelling is still controversial, suitable options for producing and identifying lactose-free products are suggested, in order to meet lactose-intolerant people’s needs.

## 1. Introduction

Lactose is the principal sugar of mammal milk, with a few exceptions (e.g., sea lions and walruses), and is the source of nourishment for newborns [1]. After weaning, mammals are not capable of digesting milk, and humans are the only species that continue to consume and digest milk daily, thanks to a phenomenon of gene–culture coevolution [2].

The main food products in which lactose can be found are dairy products, milk being their raw material, or recipes in which these products are used. Lactose can also be extracted from milk and employed as an ingredient or additive in a large variety of products. In this case, lactose is found in goods which are not directly linked to milk and for this reason it is called “hidden lactose” [3]. Regardless of its origin, lactose intake can have positive or negative effects in the human body, depending on the persistence or not of lactase-phlorizin hydrolase (LPH). This enzyme is usually located in the small intestine brush border epithelial cells and is responsible for cleaving lactose into its constituents, glucose and galactose, that can be rapidly absorbed into the bloodstream [4].

As a sugar, it has a major role as a source of energy, providing 4 kcal/g, and also having a low glycemic index [5]. It is also considered relevant in bone health due to its role in calcium absorption [6,7]. On the other hand, lactose can also induce adverse reactions. Indeed, as reported by Storhaug et al. in 2017, it is estimated that 70% of the world population suffer from lactose malabsorption. This condition makes people incapable of digesting lactose due to the non-persistence of LPH, possibly leading to the symptomatic condition of lactose intolerance (LI) [8]. Recently, an increasing number of people have become aware of this condition, resulting in a growing demand for lactose-free (LF) products suitable for their diet [9].

Unfortunately, LF product labelling is not often clear because, to date, a univocal and shared regulation in the EU about the definition and claims of LF products is lacking. Well-identified LF products suitable for the diet of people with LI are required to meet the needs of these patients. In this regard, the apposition of a recognizable mark, such as the *Lfree^®^* international certification trademark, could assist consumers with LI to make a safe and immediate choice [10].

Considering the recent growing interest concerning LI and the increasing number of LF products developed by food and drugs manufacturing companies, an update of the scientific literature about lactose utilization is required. In this regard, this review aims to investigate lactose disaccharide, from its origin and characteristics to its widespread use by modern food and drug manufacturing companies, as well as its health-related negative effects. In this way, it has been possible to hypothesize substitutions of this sugar in various food and drug products, creating LF alternatives in order to meet the needs of people with LI [1].

## 2. Physical and Chemical Characteristics of Lactose

The only natural source of lactose is mammal milk, representing its main carbohydrate, but lactose can also be extracted from it and used in various food and drug preparations as an ingredient or additive. Regarding its natural origin, lactose is synthesized in the epithelial secretory cells of the mammary gland exclusively throughout lactation. More specifically, glucose, absorbed from the bloodstream, enters the cells and is converted into uridine diphosphate-galactose (UDP-D-galactose) before being transported into the Golgi apparatus where D-glucose is located. Lactose-synthase, namely α-lactalbumin-galactosyltransferase, synthesizes lactose, joining UDP-D-galactose and D-glucose through the loss of UDP and the formation of a *β-*(1–4) glycosidic bond. Once lactose is produced, it is excreted with milk [11]. The lactose content in milk is inversely proportional to protein and fat, and varies according to the mammal species [3,12,13].

Moreover, lactose can be artificially isolated and purified for industrial uses from milk whey, which is usually a by-product in dairy industries. Before use, whey undergoes ultrafiltration in order to remove its proteins and obtain a permeate [14]. A concentration of the lactose solution is obtained by evaporation or, sometimes, by reverse osmosis. Successively, crystallization can occur by cooling down the supersaturated solution. The outcome products are recovered by decantation or centrifugation and dried in flash or fluidized beds [14,15].

Lactose is a disaccharide composed of two monosaccharides: D-glucose and D-galactose molecules joined in a *β*-1,4-glycosidic linkage [4]. Lactose can be found in two isomeric forms: *α*-lactose and *β*-lactose, according to the steric configuration of the C1 substituent group (OH and H), belonging to the glucose moiety; the two anomeric forms can be distinguished by means of their specific rotation [15].

*α*-lactose and *β*-lactose also differ in their chemical and physical characteristics, such as solubility, temperature, pH, and crystallization, leading to different features when employed as ingredients in food and drug preparations.

Solubility is an important factor concerning dissolved lactose because of its mutarotation properties. The two anomeric forms of lactose coexist in aqueous solution and are convertible between them through the open chain form of the glucose moiety of lactose, a property called mutarotation [16]. At the equilibrium, the *α*-lactose form represents 37% of the dissolved solute while *β*-lactose represents 63% [17,18].

When *α*-lactose is added to water at 20 °C it dissolves until saturation. The adding of more *α*-lactose to the solution makes mutarotation occur, so that the α-form starts converting into *β*-lactose. This phenomenon happens until an equilibrium is established between the two isomers [17,18].

Temperature has a direct influence on the amount of lactose dissolved in the solution, reaching 100 g of lactose in 100 g of water at 80 °C. The solubility of the two lactose isomers, and thus their amounts at the equilibrium, is also strongly temperature-dependent. When increasing the temperature, the *α*-form raises its solubility, shifting the equilibrium towards the *β*-form. Indeed, at 93.5 °C, the latter is the prevalent anomer in the solution [17,19,20]. However, in water, lactose does not dissolve easily compared to other simple sugars, while in liquids containing protein and fat (i.e., dairy liquids), its dissolution is slowed down due to the scarcity of available water, bound by those molecules [16].

pH does not affect the proportion of the lactose anomers at equilibrium, while it influences the rate at which mutarotation happens. At pH 5, mutarotation rate is at its minimum, but it increases at higher or lower pH values [17].

Another important aspect concerning lactose is its crystallization, which can occur when water is removed from a lactose-supersaturated solution or when temperature is reduced. Many different crystalline forms can originate from lactose, depending on the condition of the process. The main ones are the α-lactose monohydrate and *β*-anhydrous forms, from *α*-lactose and *β*-lactose, respectively. In particular, when the crystallization conditions are met, α-lactose originates its monohydrate form at temperatures below 93.5 °C. As the process continues, the equilibrium in the solution shifts from the *β*-lactose to the *α*-form, originating more and more *α*-lactose monohydrate [16], which are harder and more stable than the *β*-anhydrous form, also being less hygroscopic. Nevertheless, depending on the processing temperature, the *α*-lactose monohydrate stability can be modified, affecting its hygroscopicity. The *β*-anhydrous crystals, produced slowly at temperatures over 93.5 °C, have greater hygroscopicity and a much higher solubility compared to the *α*-lactose monohydrate crystals. These characteristics lead *β*-lactose to achieve a sweetness which is around 1.05–1.22 times higher compared to the other anomer. *α*-lactose monohydrate has the most stable crystalline form, while *β*-anhydrous crystals, in high moisture conditions, can rapidly turn into the *α*-lactose monohydrate form because of their instability. The same happens with amorphous lactose or lactose glass, originating from the rapid drying of lactose and containing both α- and *β*-lactose anomers. Being unstable and highly hygroscopic, it can be easily converted into α-lactose monohydrate by the addition of water [14,15,16,17].

## 3. Lactose in Food and Drug Industries

Thanks to its chemical-physical properties, lactose is not only found in milk, but it is often used by the food and drug industry as an ingredient or additive. It is widely used because of its easy availability and low cost as an edible raw material (mostly in the range of $794–992 per ton) [20,21,22]. The main positive and negative effects of lactose used for food and drug production, as well as its positive and negative effects on human health, are shown in Table 1 and Table 2.

### 3.1. Lactose Uses in Food Industries

Lactose can have many applications in the food industries, being exploited for the realization of different kinds of food. It is important to mention that lactose can be employed as part of an ingredient (e.g., milk, butter, yoghurt) or as an ingredient itself (e.g., cured meat, confectioneries, spray-dried preparations). The most relevant roles of lactose in food industries are summarized below, also considering the negative impact it may have [3].

#### 3.1.1. Sweetener

Sugar sweetness is measured in relation to sucrose, which is the reference sugar. Thus, a solution of 30 g/L of sucrose at 20 °C has a sweetening power of 1. Compared to sucrose, lactose sweetness ranges from 0.2 to 0.4, being one of the least sweet sugars among the main sweeteners in commerce (Table 3) [42].

Lactose plays a major role as a sweetener in confectionery, sweets, and baked goods. In confectionery, lactose is used for enhancing flavor, texture, color and stability. Its employment ranges from being an ingredient for the coating of candies, caramels, and fudges to its use in icing, avoiding cracking and chipping. Depending on the amount of lactose employed, the texture of the product can also be changed: this is noticeable in the preparation of condensed milk, where high amounts of lactose result in a grainy feel while lower amounts cause a slimy texture [14]. Because lactose sweetness is one-sixth of that of sucrose, considerable amounts can be added to food preparations, increasing the product weight without affecting sweetness as other common sugars do. Lactose can also be used to replace sucrose: the replacement of 15–20% of sucrose with lactose, in most systems, does not alter the food acceptability and makes the product more palatable, also increasing its mouthfeel and viscosity. Lactose is also used in the beer industry, especially in the production of porters and stouts. This is due to the ability of lactose in the sweetening of beer and improving mouthfeel, because brewer’s yeasts do not ferment this sugar [23].

#### 3.1.2. Browning Agent

The Maillard reaction is characterized by the reaction between amino acids and reducing sugars originating various intermediates by means of different pathways. These compounds are responsible for flavors or colors that are desirable in many food products, such as coffee or bread. The occurrence of a Maillard Reaction during storage instead is often undesirable and leads to a reduction in quality [28]. Lactose, as a reducing sugar, is involved in the Maillard reaction and, thanks to the reducing activity of its glucose moiety, the browning characteristics as well as flavor and odor in baked goods are enhanced. For this reason, lactose is used in sweet or savory bakery goods to obtain a golden crust and suitable toasting quality [21,29]. During the leavening of the dough, sugars are usually fermented by microorganisms, but some of them do not use lactose as a source of energy, so that it remains in the final mass. This not only leads to the enhancement of the browning of the loaf during its baking, but also that of its slices when toasted [23]. Nevertheless, lactose vulnerability to browning can also result in an excessive and undesired color-change in dried milk and dried whey powder during storage.

Furthermore, lactose is involved in baking as it increases the volume of loaves and biscuits. Moreover, thanks to lactose hygroscopicity, the tenderness of baked products is improved, resulting in a better mouthfeel and texture of the baked goods, prolonging their shelf-life [14].

#### 3.1.3. Encapsulating Agent

Encapsulation is a method by which a mixture of ingredients is coated with a material that acts as a barrier, protecting the core. Lactose is a common encapsulation agent or carrier during spray drying because of its relatively high glass transition temperature when compared to other monosaccharides/disaccharides [25]. Lactose can be used as an encapsulant agent for the spray drying technique, having a bland flavor, discrete solubility, and low viscosity, even if it is rarely used on its own. It is used in the preparation of a wide variety of dried products, ranging from foods, such as milk, cheese, whey, and ice cream mixes, to drugs. When the solution to be dehydrated is quickly dried, the added lactose assumes its amorphous form, tending to coat the solute particles [33]. Furthermore, given lactose hygroscopicity, covering food particles with glass lactose enables the formation of a crystalline capsule of *α*-monohydrate when moisture is absorbed during storage. The crystalline structure keeps the coated food particles separated and facilitates their dispersion, especially in powdered foods. For this reason, in certain conditions, lactose is also considered a free-flowing agent [14].

#### 3.1.4. Anti-Freezing Agent

Lactose can also play a fundamental role as an anti-freezing agent, for example in the production of ice cream. Freezing is indeed a crucial phase in ice cream making since it deeply influences the organoleptic characteristic of the final product. Depending on the quantity of solutes (e.g., lactose and salts) in the ice cream mix, the freezing temperature changes and the more lactose is added to the ice cream preparation, the lower is the temperature needed to properly freeze it. This phenomenon influences the hardness of the ice cream, because the addition of lactose in the preparation produces a softer ice cream than an ice cream with less lactose at the same serving temperature. On the contrary, when lactose is present in high amounts, supersaturation of the solution may occur, and this favors lactose crystallization, resulting in a coarse or icy texture of ice cream and reducing the shelf-life of the product [31].

#### 3.1.5. Shelf-Life Extender

Baked goods are staple foods that undergo changes during their shelf-life which impair their quality. Staling is a phenomenon that commonly determines organoleptic impairment [36,37]. The addition of lactose during the preparation of baked goods increases the possibility of extending their shelf-life thanks to its ability to adsorb volatiles and coloring agents. In particular, lactose is able to influence consistency, such as the viscosity and texture of the product, and indeed it contributes to softness and lower humidity, reacting with proteins and also creating an extension of shelf-life [30].

#### 3.1.6. Fermentation Substrate

As a potential fermentation substrate, lactose can be either part of milk used to obtain the final product, such as in yoghurt and kefir, or it can be employed as an additive, such as in cured meats. Lactose can indeed be added and used by the selected starter microorganisms as a source of energy for their fermentation activity in cured meats to obtain specific organoleptic properties. Lactobacilli, lactic acid bacteria used for processing meat, split lactose into galactose and glucose and convert them into lactate, thus slowing down the metabolizing of the sugar and the acidification of the product. Acidity causes a decrease in the water-binding capacity of proteins, accelerating the drying and, as a consequence, shortening the processing time [38].

In fermented dairy products (e.g., yoghurt and kefir) lactose is instead a part of milk, and is metabolized by the added starter microorganisms. In particular, S. thermophilus and L. delbrueckii subsp. bulgaricus strains are specifically used in yoghurt production, causing a decrease in pH and thus the coagulation of milk proteins [39].

### 3.2. Lactose Uses in Pharmaceutical Formulations

Drugs are composed of active pharmaceutical ingredients (APIs), commonly considered the most important substances in the formulation because of their pharmaceutical action, and excipients, usually critical for drug bioavailability. Lactose is one of the most commonly used excipients in the pharmaceutical industry, owing to its physical and chemical properties such as chemical inertia, stability, and non-toxicity, besides being moderately priced [25,26,27].

Considering the organoleptic characteristics of lactose powder, i.e., it being white and odorless and having a sweet taste, its acceptance as a component in pharmaceutical formulations is notable. There are many ways to use lactose as an excipient and different pharmaceutical forms contain it; indeed it is found in about 20% of prescription medicines and in 6% of over-the-counter medicines. For instance, lactose can be used as a diluent in tablets, lozenges, capsules, and powder for intravenous injections [35]. Tablets are the most common pharmaceutical form on the market containing lactose. In order to ensure adequate product processability throughout manufacturing, a precise volume of powder is needed to create tablets. Therefore, when the quantity of API is low, diluents, such as lactose, are necessary to adjust the mass of the solid dose. Sometimes diluents, also referred to as fillers, may constitute up to 90% of the total dosage weight. Lactose can act as a soluble diluent in formulations due to its excellent flowability and compressibility as well as its rheological properties [25,35]. In tablets, lactose powder can be used in different sizes of granulation and crystal forms in order to modulate its properties in the formulation. Moreover, lactose particles of the same size-range produce granules of higher porosity, which will improve the drug’s dissolution after being compressed. In this context, *β*-lactose crystals provide better compressibility properties and superior tensile strength values in comparison to *α*-lactose [27,43,44].

In Dry Powder Inhaler (DPI) formulations, lactose is often used as a carrier. The API used in DPIs should be aerodynamic and smaller than 5 µm, as well as having rheological properties such as flowability, stability, and uniformity. *α*-lactose monohydrate has all of these performing characteristics along with a well-established safety profile and compatibility with most available low-molecular-weight APIs [45,46]. Unfortunately, lactose carrier particles are too large to penetrate into the deep parts of the respiratory system, so that most of the lactose deposits in the oropharynx. In this case, if the molecule is swallowed, lactose can reach the gastrointestinal tract, giving rise to intolerance effects in case of LI [47]. Furthermore, lactose can work as a cryo/lipoprotective excipient during the freeze-drying process thanks to its adsorption properties, which can protect drugs against moisture [32]. Lactose is also used as a cross-linker in hydrogel of gelatin, obtaining a rigid layer able to provide mechanical strength and protection to the dressing [40].

In conclusion, the lactose content in drugs ranges from 100 to 200 mg, usually not exceeding 400 mg per tablet or capsule; however, this amount can have undesirable effects in patients who suffer from LI. However, it is very difficult to predict how the amount of the lactose intake changes depending on API doses or on the digestive process, due to different rates of gastric emptying, pH, and intestinal motility [25].

### 3.3. Undesirable Properties of Lactose

Lactose utilization can also result in undesirable effects during the preparation of food or in the final product [21].

#### 3.3.1. Solubility

One of the undesirable properties of lactose utilization is its low solubility, which can result in crystallization, giving a gritty and sandy mouthfeel in the final product. Usually, in supersaturated solution, sugars tend to crystallize, also forming big agglomerates, depending on the process condition [23]. The tendency of sucrose and lactose to form big crystals can be influenced by the relative concentration of other sugars in the solution. In particular, to avoid the sandy mouthfeel caused by their crystallization, lactose is usually added to the formulation, delaying the process [21]. In this context, in confectionery, the adding of lactose to a sucrose solution progressively induces the formation of smaller crystals, leading to a smoother crystalline mass [22]. In condensed milk, the typical graininess caused by lactose crystallization is avoided by a rapid cooling of the solution and by seeding with powdered *α*-lactose monohydrate. The seeding technique ensures that lactose crystals are 10 μm or less [24].

#### 3.3.2. Stickiness and Caking

Caking can be defined as a phenomenon in which a low-moisture, free-flowing powder is transformed into lumps and then into an agglomerated solid, losing its quality and function. Among the substances studied, amorphous lactose and dairy milk powder can be found [33,34]. In particular, low-molecular-weight sugars are known to cause caking during storage [48]. Lactose powders can undergo chemical changes due to humidity and/or exposure to high temperature. Under these conditions, amorphous or crystalline lactose absorbs water, becoming progressively stickier and resulting in its plasticization [27,43]. The glass transition temperature of anhydrous amorphous lactose has been indicated as 101 °C or 115 °C, whereas that of skim milk solids has been reported as 92 °C [16]. When lactose is used as a coat for powdered substances, when water is absorbed, its plasticization can therefore result in a lactose glass transition, creating viscosity problems and stickiness [31].

## 4. Negative Effects of Lactose on Health: Lactose Intolerance

As previously stated, lactase-phlorizin hydrolase (LPH), a *β*-galactosidase belonging to the class of hydrolases, is the enzyme responsible for lactose digestion [49]. LPH is essential for the nourishment of neonatal mammals because lactose itself cannot be absorbed and needs to be cleaved in the intestinal lumen before proper digestion. Intestinal LPH activity increases until week 34 from conception and reaches its peak around the first months after birth. Afterwards, LPH activity gradually decreases because of a lower lactase gene expression, resulting in the lactase non-persistence condition (LNP) [12]. In humans, a small portion of the population maintains LPH activity throughout adulthood; however, this is not common in other mammals. In case of lactase persistence (LP), instead, the activity of LPH continues, being associated with several single nucleotide polymorphisms (SNP) in the lactase gene [50].

It is estimated that about 70% of the adult population worldwide has a limited expression of the lactase enzyme, with a large variation between different regions and countries [8]. This condition can be caused by different reasons, such as the above-mentioned genetically determined LNP or the presence of other gastrointestinal disorders that damage the gut brush border where LPH is located [4]. In both cases, lactose cannot be digested (lactose maldigestion) and thus absorbed (lactose malabsorption), leading to its fermentation by the intestinal microbiota. Lactose maldigestion and lactose malabsorption can ultimately lead to the onset of a clinical condition characterized mainly by the presence of gastrointestinal symptoms, defined as LI [3]. This condition causes gas production and therefore flatulence, meteorism, and abdominal distention, while an over-production of short-chain fatty acids produces acidification and irritation of the gastrointestinal mucosa, which results in pain and diarrhea or constipation [41].

In addition to the above-mentioned symptoms, which are closely related to the gastrointestinal tract, there is the possibility of developing the less typical symptoms of malabsorption, which are usually extraintestinal. These could be headache, joint and/or muscle pain, eczema, mouth ulcers, heart palpitations, skin lesions, and increased urination [3]. The clinical manifestation variation among individuals with LI is probably due to the psychosomatic component and in the composition change of the gut microbiota microorganisms that lead to a more or less significant production of lactase [31]. Regardless of symptom severity, LI impacts on the quality of life and nutrition of people suffering from this condition. The anxiety of feeling sick after the ingestion of food products containing lactose can lead to the worsening of symptoms. In addition, this anxiety makes lactose-intolerant people follow a restrictive diet to avoid not only food containing lactose, but also those usually associated with gastrointestinal adverse effects, such as bloating, for the fear of experiencing the symptoms [1,51].

## 5. Regulation on Lactose Labelling

### 5.1. Food and Supplements

**Figure 1:**
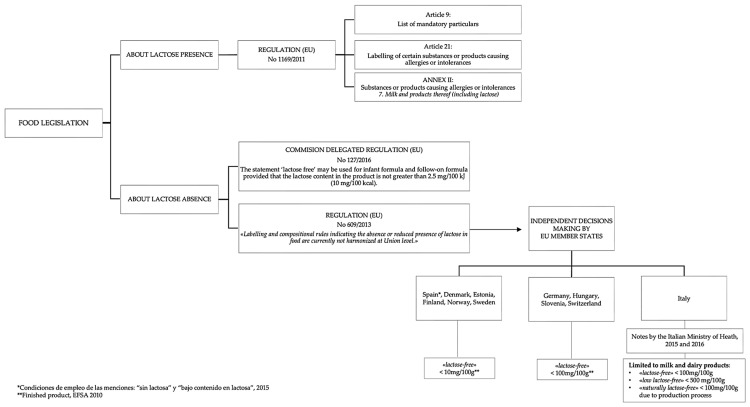
Overview of regulation about lactose in Europe [EFSA Panel on Dietetic Products, Nutrition and Allergies (NDA) [58,59].

In order to address the needs of people with LI, the EU by Regulation (EU) No. 1169/2011 requires the provision of information about allergen presence in food products (Figure 1). This regulation identifies 14 substances or products causing allergies or intolerances; among them, milk and its derivatives, lactose included, are reported [52]. Despite this, in the EU, declarations about the absence or the decreased amount of lactose in foods are not currently harmonized. European legislation assessed the urgency for paying more attention to allergen information on food labels with Regulation (EU) No. 609/2013, integrated by Commission Delegated Regulation (EU) 2016/127, allowing the utilization of the “lactose-free” claim only for food products with a lactose content less than 10 mg/100 kcal addressed to children. Since then, no other rules at Union level have been issued on the topic [53,54].

The Italian Ministry of Health has expressed opinions on the matter with the aim of providing adequate information to companies and consumers. In 2015, an official note explained that the indication “lactose-free” may be used for milk and dairy products with a residual lactose content of less than 0.1 g per 100 g or mL, and “reduced lactose content” may be used if the disaccharide residue is less than 0.5 g per 100 g or mL in milks and fermented milks, pending harmonization of the issue at the European level. A “naturally lactose-free” indication may be used for products with no milk ingredients in conformity with Art. 7 of Regulation (EU) No. 1169/2011, for which labels should not report misleading information. In particular, they should not emphasize the presence or absence of certain ingredients when all similar foods possess such characteristics [55,56].

A note from the Italian Ministry of Health was issued in 2016 with the aim of defining “naturally lactose-free” products, integrating the previous. It reports that dairy products with a lactose content that is naturally reduced thanks to their typical manufacturing process may use the “naturally lactose-free” indication if their residue is less than 0.1 g per 100 g [57].

Given this, it can be clearly understood how difficult it is both for food manufacturing industries to label their goods and for consumers to understand the many indications they find on the products. It is also important to mention that it is possible to find milk, dairy products and other food products on the market declared as “lactose-free” having a lactose residue of less than 0.01 g per 100 g. This value has been used as a suitable reference for the “lactose-free” indication by several countries including Finland, Norway, Sweden, Denmark, and Estonia according to the EFSA Panel on Dietetic Products, Nutrition and Allergies (NDA) [58] (Figure 1).

Nowadays, there is therefore not a universal law regulating the production and commercialization of “lactose-free” products. Moreover, a cut-off value for lactose content, establishing whether a product can be labelled as “lactose-free”, is missing and the definition of “traces” is not regulated yet [3]. The result is the proliferation of many food products with different indications on the label and the consequent confusion of consumers. Indeed, not all consumers are aware of the specific lactose amount contained in food and spend a lot of time reading and understanding the labels of LF products when they do grocery shopping. Several characteristics of labels have been identified as responsible for increasing the effectiveness of the front-of-package systems. This includes the use of recognizable symbols that are easy to understand, combined with simple text descriptors [3,60]. In Italy, the lactose-intolerant patients’ association, AILI (Associazione Italiana Latto-Intolleranti) supported ELLEFREE Srl in the creation of the first internationally registered symbol that identifies and certifies lactose-free and milk-free products, named Lfree^®^. This mark can be an adequate visual symbol and tool for identifying food products having the lowest detectable amount of lactose (0.001 g per 100 g) by modern analytical techniques. Therefore, the Lfree^®^ mark could be the right guide for consumers with LI to easily recognize LF products suitable for their condition [3].

### 5.2. Drugs

Drug-labelling legislation is regulated by the European Medicines Agency (EMA), which manages all the medicinal trade authorization at the European level for member states which participate in it. EMA has the law force of legislating drugs development for its member states and their market, and it is responsible for coordinating the scientific resources. According to EMA, all excipients of drug should be listed in the Summary of Product Characteristics (SmPCs), regardless of their doses. The SmPCs is a legal document approved as part of the marketing authorization and represents the basis of information for healthcare professionals. The SmPCs describes not only the quantity of excipients contained in the pharmaceutical formulation, but also the potential exposure of the patient on the basis of the daily intake of the drug. Package leaflets are based on SmPCs information, and it is specified that excipients with recognized action or effect need to be declared on the labelling. In the package leaflets of injectable, topical, or eye preparations, all excipients must be listed, as reported by EMA in 2003 [61].

Due to its possible recognizable negative effects, lactose is specifically reported as an excipient in the current effective version of the Annex to the European Commission guideline on ‘Excipients in the labelling and package leaflet of medicinal products for human use’, and thus a statement should be present on the label of each medicinal product that contains it [62]. Since industries are required to list the excipients but they are not required to quantify their amount, doctors and pharmacists may not know how much of the excipients and therefore of lactose is contained in the prescribed drugs. Such information should be found in SmPCs, also reported in the compendium of medicines (MC) or in its electronic version (ECM). In this regard, as reported by Eadala et al. in 2009, the information about lactose quantities should be displayed in a prominent place on the labels of drugs, such as on the front-of-pack [25]. Preferably, a well-recognized and univocal symbol such as Lfree^®^ could guide doctors and pharmacists in prescribing LF drugs.

When the substitution of drugs in a product is desired, e.g., the replacement of lactose with a substitute, a demonstration about the bioequivalence of the two drug products is required. According to the EMA guidelines, two drugs containing the same pharmacological substances are considered bioequivalent if, after administration at the same dose, their bioavailability (speed and extent of drug absorption) is within acceptable predefined limits [63,64,65].

## 6. Development of LF Products

To date, the main treatment for lactose intolerance is to exclude or reduce products containing lactose from the diet. In this way, the respite of the manifestation of the symptoms, which impairs the health-related quality of life, occurs. Lactose can be present in food as a natural part of an ingredient, such as in milk and dairy products, or as an additive by itself, for example in bakery products or cured meats. Recently, food industries have been able to use LF alternatives to obtain product lines specifically addressed to consumers with LI. The LF market, meeting the needs of people with LI by the development of innovative LF products, is steadily increasing, reaching a growth in sales of +7.8% from 2019 to 2020 [9].

### 6.1. Substitution of Ingredients Containing Lactose

In order to produce LF products, suitable ingredients should be considered. Many ingredients containing lactose can be easily replaced by LF alternatives, as reported in Table 4.

Some of these ingredients are specifically produced in order to diminish their lactose content, e.g., “delactosed” products, other ingredients lose lactose thanks to their natural production process, while others do not contain lactose, e.g., flour and eggs. Additionally, a current way to meet the needs of this sector is the development of beverages based on plant-based extracts, called “plant milk substitutes”, such as soy, rice, corn, nuts, almonds, among others, although these products are poorer in terms of nutrition and sensory acceptability [66,67,68].

It has to be noted that suitable products for a LF diet have to contain a lactose amount that does not cause the adverse symptoms typical of the LI condition. Since a LF recipe can include different LF ingredients, the amount of lactose in the final product represents the cumulative effect of lactose contained in each of them. This is an issue that needs to be taken into account for “delactosed” and naturally lactose-free products because of their possible residual lactose amount.

LF ingredients for LF products are represented by the following categories:

- **“Delactosed” ingredients**: This group includes milk and dairy products subject to enzymatic treatment that breaks down lactose into glucose and galactose, which assures a LF final product under certain conditions.
  Lactase is the enzyme used by food manufacturing companies to produce milk-based LF products. This enzyme is widely found in nature and can be isolated from plants, yeasts, fungi, bacteria, and animals. The production of LF milk and its derivatives by the hydrolysis of lactose was developed in 1970, when the first β-galactosidase became commercially available [69].
  The use of soluble lactase for the production of LF milk can be effective using two different methods: “in batch”, with the addition of a relatively high amount of lactase to raw or heat-treated milk before an incubation of about 24 h [70], and “in pack or aseptic”, involving lactose hydrolysis also during product storage [71]. The quality of low-lactose milk is affected by the side proteolytic activity of the lactase used in the production process [72].
- **Naturally lactose-free** (**NLF**) **ingredients**: This category identifies products, obtained from milk transformation, in which lactose is naturally reduced thanks to their typical manufacturing process. Cheeses are the main representative of this group and synergic factors work together to reduce lactose content. As reported in Facioni et al. (2021), some of the main parameters involved are the microbial composition of the starter culture, curd processing, and, lastly, ageing. The standardization of the cheese production process plays a crucial role in determining obtaining the same lactose residue among the final products of the process, and this also assures its reproducibility. PDO cheeses are the most suitable category of products that respect the above-mentioned requirement because they comply with technical specifications reporting agreed cheese production rules. It has been shown that, in specific cases, a NLF cheese is obtained within a few months from production. Despite this, it is crucial not only to study case-by-case situations, but also to perform analytical validation of the final product to assess residual lactose content, because of the variations allowed within the product specifications [73].
- **Ingredients not containing lactose**: This group includes ingredients which do not contain lactose, nor milk or dairy products, because of their specific natural composition. Some examples are water, flour, vegetable oil, eggs, vegetables, meat, and fish, together with processed ingredients, mainly plant-based beverages such as those obtained from rice, soy, oats, coconuts, nuts, almonds, cashews, hemp, etc.

### 6.2. Substitution of Lactose as an Additive

Lactose alone can be used as an additive to provide certain properties to the food or drug in which it is employed. To date, specific studies about the replacement of lactose with a suitable substitute having the same or similar characteristics are scarce. Nonetheless, in the present study, hypotheses are proposed to focus on the issue and raise awareness regarding the lack of proper information on the topic.

#### 6.2.1. Sweetener

As stated in Section 3.1.1, sugar sweetness is measured in relation to sucrose. Lactose has a sweetening value ranging from 0.2 to 0.4 [42]. Therefore, replacing sugars with comparable sweetening power could be maltose, lactitol, galactose, and raffinose. In this context, lactitol (E966) is a disaccharide with a limited sweetening power compared to the other polyols, so that it is usually employed coupled with intense sweeteners. The human body is not capable of metabolizing lactitol, so that it does not contribute to caloric intake. It is characterized by giving a fresh aftertaste when consumed, being responsible for conferring different kinds of sweetness to food. Given its scarce sweetness, it is also used to increase food volume without affecting its karyogenicity. Lactitol finds its employment in foods like chocolates, baked goods, bubble gums, and ice creams [74]. Sucrose, sucralose, and stevia extract have also been evaluated as sweeteners in lactose-free chocolate-flavor frozen dessert formulations, showing differences in milk flavor, a bitter taste, bitter residuals, and melting. In particular, in comparison with sucrose, the use of stevia extract produced a bitterer taste that inhibited the perception of milk flavor, although the acceptability was not significantly affected. These differences were less noticeable with the use of sucralose [75].

#### 6.2.2. Browning Agent

Reducing sugars can have different relative reactivity in the Maillard reaction. The numerous factors involved, for example concentration and nature of the amino and carbonyl compounds, water activity, heating time, temperature, and pH, lead to many outcomes in browning products. It is generally recognized that the relative reactivity of different sugars is highest in pentoses, followed by hexoses, disaccharides, and aldoses, while chetoses are the least reactive. Among them, ribose, xylose, arabinose, glucose, and fructose can be used to induce the reaction [62].

#### 6.2.3. Encapsulating Agent

Encapsulation is a common process of coating especially used for milk fat, ice-cream mixes, vegetable oils, and other fats. Various encapsulant agents can be used for obtaining a stable emulsion before the spray drying process, and choosing the right agent is critical. Indeed, the powder flowability, shelf-life, and mechanical stability depend on the balance between the components of the original liquid mix. To date, despite encapsulated fat being susceptible to many quality issues, there are no specific and exhaustive studies about the topic. Solutions have been proposed regarding butter powders, where the solids-not-fat matrix may consist of milk protein products such as nonfat dry milk, sodium caseinate or whey proteins, various sugars, starches, gums, emulsifying agents, and/or sodium citrate. Other encapsulant agents can be milk proteins such as sodium caseinate or whey proteins, carbohydrates such as starch, maltodextrins or corn syrup solids, or waxes and gums such as gum arabic [76,77].

#### 6.2.4. Anti-Freezing Agent

Freezing point depression is a function of both the concentration of all the solutes and their molecular weight. The removal and substitution of lactose as a freezing agent from the preparation can be obtained by its hydrolysis, using dairy products as a base. In this way, this sugar is reduced in ice cream preparations, being substituted by its hydrolysis products, glucose and galactose, as anti-freezing agents. This substitution doubles the freezing point depression of an equal concentration of lactose and an increase of the relative softness at storage or retailing temperatures, but also causes a higher rate of ice recrystallization. Given the major sweetness of lactose-hydrolyzed ice cream compared to its unhydrolyzed version, a reduction in sucrose has to be considered to obtain the optimal formulation both for sweetness and freezing point depression [31].

#### 6.2.5. Shelf-Life Extender

The bakery industry has been working to identify treatments which allow an extended shelf-life for bread [36,37]. An extension of shelf-life can be obtained using other sweeteners such as stevia, specifically its water extract. This sweetener can increase the shelf-life of bread to 5–7 days. In particular, stevia improves the quality of products, increasing the biotechnological properties of yeast and the maturity of semi-processed products [78].

#### 6.2.6. Fermentation Substrate

Recently, companies have decided to eliminate or replace lactose in their processing, so to date, most cured meats do not contain lactose. Regarding heat-treated cured meats, such as “prosciutto cotto” (namely cooked ham), lactose has been added as a part of caseinates. These milk proteins are usually employed as emulsifiers due to their influence on water retention increase, and therefore they also influence the final yield of the product. Replacing caseinates with other animal-origin proteins such as collagen-origin or plasmatic-origin proteins is a way to maintain the yield of the product while removing lactose from it. On the other hand, for fermented cured meat, such as fermented sausage, other simple sugars can be employed, although to the best of our knowledge, such studies are not the subject of recent literature [79,80].

### 6.3. Substitution of Lactose as a Drug Excipient

As reported in Section 3.2, lactose is an excipient, more specifically a filler, having excellent physical and chemical properties, so that its replacement in drug formulations could be challenging. Indeed, in medicines, the excipient is the substance that is responsible for the absorption and the effectiveness of the drug. The role of lactose in drugs can be played by other excipients, such as sucrose, glucose, mannitol, starch, cornstarch, and microcrystalline cellulose [35]. Sucrose, glucose, and mannitol are usually employed to replace lactose in lozenges that are the result of a drying process rather than compression, so it is not necessary for the excipient to have an excellent flowability and compressibility, as is required for lactose. Sucrose can also be used as a lactose substitute in tablets and granulates, whereas mannitol can be a substitute in solutions for injections and sublingual tablets. Nonetheless, both excipients may cause adverse effects [35,61].

Another possible replacement of lactose can be starch or cornstarch, which can be used to produce tablets, due to its adhesive, stabilizing, and film-forming properties, even if it is more suitable as a disintegrating excipient in tablets than as a filler. Moreover, cornstarch is quite insoluble and characterized by high swelling capacity. However, cornstarch is used in combination with lactose as a carrier in spray-drying preparations, and not as a substitute, to merge the compaction and binding properties of α-lactose with the disintegrant characteristics of cornstarch [27,35].

Microcrystalline cellulose can also be used to replace lactose, since tablets containing microcrystalline cellulose are hard but dissolve quickly. Microcrystalline cellulose is used as a diluent and a binder for capsules and tablets, both in wet granulation and in direct compression, and as a lubricant and a disintegrant in compression.

In conclusion, even if lactose is the most versatile of the excipients, when necessary, there are substances that can replace it in various pharmaceutical forms. The various substitutions however may lead to a change in the bioavailability of the drug and its chemical stability. This implies that specific investigations of the excipient-API combination are necessary and that further studies in pharmaceutical chemistry are required.

## 7. Conclusions

Lactose is the principal milk sugar, and it is often employed in the formulation of food and drug products due to its peculiar chemical-physical characteristics. Given its versatility, this sugar finds its way in formulations as an ingredient itself or as part of a dairy ingredient. Considering the increasing awareness about the condition of LI, the demand for LF products is increasing, so that food and drug industries are implementing new technologies to develop LF options meeting lactose intolerant people’s needs. Most of the worldwide population seems to suffer from LI, a symptomatic condition that makes people unable to correctly digest lactose and thus the products that contain this sugar. A comprehensive scientific literature about lactose uses and its substitution in food and drug products is lacking. In this context, a deeper insight is required to provide suitable LF products to people with LI. The main properties of lactose were reviewed and possible alternatives for food and drug preparation were hypothesized. Given the implication of these products in fulfilling a specific nutritional need, their immediate and clear identification is essential. In this regard, the *Lfree^®^* certification mark could be a useful labelling tool to identify and guarantee LF products with a lactose residue of less than 10 mg/kg.

## Figures and Tables

**Table 1:** Main positive and negative effects of lactose in food and drugs.

INDUSTRY USE							
Food				Drug			
Positive Effects	References	Negative Effects	References	Positive Effects	References	Negative Effects	References
Sweetener	[14,23]	Solubility	[21,23,24]	Inert	[25,26,27]	Health-related effects	[25]
Browning agent	[14,21,23,28,29]	Stickiness	[30,31,32]	Stable	[25,26,27]		
Encapsulating agent	[14,28,33]	Caking	[33,34]	Non-toxic	[26,27]		
Anti-freezing agent	[31]			Diluent	[35]		
Shelf-life extender	[14,36,37]			Flowable	[25,35]		
Fermentation substrate	[23,38,39]			Compressible	[25,35]		
				Excellent rheological properties	[25,35]		
				Cryo-lipoprotector	[32]		
				Cross-linker	[40]		

**Table 2:** Main positive and negative effects of lactose on human health.

HEALTH-RELATED EFFECTS				
Positive Effects	References	Negative Effects		References
4 Kcal	[5]	Gastrointestinal symptoms	Abdominal distention	[3,41]
Calcium absorption	[6,7]		Constipation	[3,41]
Health of bones	[6,7]		Diarrhea	[3,41]
Low glycemic index	[5]		Nausea	[3]
Low cariogenicity	[5]		Meteorism	[41]
			Flatulence	[41]
		Extraintestinal symptoms	Skin rashes	[3]
			Headache	[3]
			Increased urination	[3]
			Joint and/or muscle pain	[3]
			Heart palpitations	[3]
			Mouth ulcers	[3]

**Table 3:** Main sugars and their sweetening power compared to sucrose, modified from [42].

SUGAR	SWEETENING POWER Compared to Sucrose (=1)
Advantame	37,000
Neohesperidin	1500–2000
Aspartame	200
Fructose	1.1–1.15
Sucrose	1
Glucose	0.75
Mannitol	0.6
Sorbitol	0.6
Isomaltose	0.55
Maltose	0.4
Lactose	0.2–0.4
Lactitol	0.35
Galactose	0.3
Raffinose	0.2

**Table 4:** Ingredients containing lactose and their possible substitutions.

RAW MATERIALS Contening Lactose	POSSIBLE ALTERNATIVES
Milk	Lactose-free milk Vegetable drink (soy, rice, spelt, almond, coconut, oats, etc.) Water Lactose-free yogurt
Butter	Butteroil Lactose-free butter Ghee Vegetable butter (cocoa, shea) Vegetable oil (olive, sunflower, corn, safflower, etc.) Margarine 100% vegetable Vegetable cream
Dried milk	Lactose-free dried milk Vegetable dried milk
Cheese	Naturally lactose-free cheeses “Delactosed” cheeses Plant-based alternatives
